# Digestibility of Conventional and Novel Dietary Lipids in Channel Catfish *Ictalurus punctatus*

**DOI:** 10.3390/ani13091456

**Published:** 2023-04-25

**Authors:** Andrew Maina, Rebecca Lochmann, Steven D. Rawles, Kurt Rosentrater

**Affiliations:** 1 Catalent Pharma Solutions, 160 N. Pharma Drive, Morrisville, NC 27560, USA; amaina1080@hotmail.com; 2 Department of Aquaculture and Fisheries, University of Arkansas at Pine Bluff, Mail Slot 4912, 1200 N. University Dr., Pine Bluff, AR 71601, USA; 3 Harry K. Dupree Stuttgart National Aquaculture Research Center, Stuttgart, AR 72160, USA; 4 Department of Agricultural and Biosystems Engineering, Iowa State University, Ames, IA 50011, USA

**Keywords:** channel catfish, lipids, fatty acids, digestibility, feed formulation

## Abstract

**Simple Summary:**

The digestibility of ingredients in fish diets must be known to use the least-cost feed formulation. However, there is very little published information on lipid (fat) digestibility in fish. With this information, commercial feed producers can choose lipids based on high utilization by the fish, as well as cost. We conducted a feeding trial with catfish fed diets with different lipids: soybean oil, soybean oil with conjugated linoleic acids, catfish offal oil, flaxseed oil, menhaden fish oil and poultry fat. After feeding, fish feces were collected for nutrient analysis and compared to the lipid content of the diets. Lipid and fatty acid digestibility were high overall for all of the lipids tested. However, the digestibility of certain fatty acids was different from overall lipid digestibility. This information can be used to choose the best lipids to meet catfish needs, enhance healthy fats in the fish for human consumers, and produce a cost-effective feed.

**Abstract:**

Lipid and fatty acid digestibility is presumably high in Channel Catfish, but data is lacking. We determined the lipid and fatty acid digestibility of traditional and alternative dietary lipids in Channel Catfish to inform lipid choice for commercial diets. Six diets contained 4% of different lipids: soybean oil (SBO), soybean oil containing conjugated linoleic acids (CLA-SBO), catfish offal oil (COO), flaxseed oil (FXO), menhaden fish oil (MFO) and poultry fat (PF). Diets were fed to Channel Catfish (150–200 g) maintained at 26.5 °C in each of six 110 L aquaria. Six hours post-prandial, feces were collected for analysis. Total lipid, crude protein and fatty acids of lyophilized feces were analyzed, and apparent digestibility coefficients (ADCs) were calculated. ADCs of lipid, saturated and monounsaturated fatty acids, linoleic acid and protein digestibility were similar among diets. CLA isomers (*cis*-9, *trans*-11 (84.1%) and *trans*-10, *cis*-12 (90%)) in the CLA-SBO diet were highly digestible. Oleic acid digestibility was highest in the PF diet. ADC was high for α-linolenic acid in the FXO diet, and for arachidonic acid and n-3 LC-PUFA in the MFO diet. Overall, total lipid digestibility was high, but ADCs of individual fatty acids differed by source.

## 1. Introduction

The catfish industry is the largest commercial aquaculture industry in the USA, with producer sales of USD 447 in 2022 [1]. Feed costs have increased steadily over time, prompting a search for cheaper alternative ingredients. However, exchanging conventional dietary lipid sources for alternative lipid sources in fish feeds to gain an economic or sustainability advantage might also affect lipid and nutrient digestibility [2]. Lipid and fatty acid digestibility studies allow screening of new ingredients for nutrient bioavailability to the fish [3]. Digestibility in catfish typically does not include lipid sources [4], because it is assumed that lipid and fatty acid digestibility are high. Due to the wide variety of lipid sources used in channel catfish feeds [5,6,7,8], there is a need to understand the digestion and absorption of dietary nutrients when using different dietary lipid sources. This information could assist feed producers in considering different lipid sources for inclusion in commercial Channel Catfish feed formulations.

Animal fats are high in saturated fatty acids, while plant oils are rich in polyunsaturated fatty acids (PUFA) and monounsaturated fatty acids (MUFA). The dietary lipid source [9] and fatty acid profile affect the digestibility of dietary lipids [10]. The degree of saturation of fatty acids [11], fatty acid chain length [12] and saturated fatty acid inclusion level in the diet [13] all affect lipid digestibility.

Diets for Channel Catfish are formulated assuming that there are no differences in the digestibility of different lipid sources. Lipids must meet the essential fatty acid (EFA) requirement of the fish [4]. Channel Catfish require both n-3 and n-6 PUFA in their diet [14,15]. However, the constituent fatty acids of the dietary lipids also affect the fatty acid composition of the fish, which affects the nutritional value of the fish, as well as organoleptic and storage properties [16,17]. Marine fish oils are not major components of commercial Channel Catfish diets, which results in low levels of n-3 long-chain polyunsaturated fatty acids (n-3 LC PUFA) in catfish fillets. These fatty acids, including eicosapentaenoic acid (EPA) and docosahexaenoic acid (DHA), are considered beneficial for human health [18]. Marine fish oils were minimized or eliminated from commercial Channel Catfish feeds due to their cost and negative effect on the peroxide values of the feeds. In addition, catfish can synthesize n-3 LC-PUFA from 18-carbon precursors, so preformed n-3 LC PUFAs are not required in the diet. Alternative lipid sources are available that can support fish performance while supplying other healthy fatty acids, such as conjugated linoleic acids (CLAs).

Lipids containing CLAs have been used successfully in diets for Channel Catfish [17,19,20,21]. The CLAs have been implicated in mitigating cancer, heart disease, obesity, diabetes and bone pathologies in animal models, as well as in vitro studies [22]. Similar to other dietary fatty acids, the CLAs accumulate in the fish fillet. However, their use does not increase diet cost as much as fish oil, or reduce sensory characteristics of fish compared to catfish fed diets with fish oil [17]. Differences in the digestibility of traditional and novel oils should also be considered in formulating commercial catfish feeds. In this study, the digestibility of CLA-SBO was compared to other practical lipids to determine the nutrient bioavailability of the lipid sources. The null hypothesis is that there would be no differences in the digestibility of lipids or fatty acids among lipid sources.

## 2. Materials and Methods

### 2.1. Diet Preparation

A basal diet (sinking pellet) was formulated to meet the nutrient requirements of Channel Catfish [4] (Table 1). The diets were prepared by Dr. Kurt Rosentrater at Iowa State University (Ames, Iowa). The proximate and fatty acid composition of the diets are shown in Table 2 and Table 3, respectively. Yttrium oxide (Yt, 0.5%) was included as the inert marker. The lipid sources tested included some that are commonly used in Channel Catfish diets as well as some novel sources. The tested lipids and their distinctive fatty acids were: soybean oil (SBO; 18:2n-6), soybean oil enhanced with conjugated linoleic acids (CLA-SBO; CLA isomers and 18:2n-6), catfish offal oil (COO; 18:1n-9), flaxseed oil (FXO; 18:3n-3), menhaden fish oil (MFO; n-3 LC-PUFAs) and poultry fat (PF; 18:1n-9). Collectively, the lipid sources contrasted in their fatty acid makeup. Ethoxyquin (0.0125%) was added to the supplemental lipid in all diets to limit lipid peroxidation.

All ingredients were milled with a hammer mill (Glen Mills, Clifton, NJ) and thoroughly homogenized in a mixer (Kobalt, Greenfield Center, NY). Each blend was adjusted to a desired pre-extrusion moisture content of ~45% by adding appropriate quantities of water, then mixed again for 15 min to ensure complete moisture distribution. The extrusion processing of each blend was performed using a single screw commercial extruder (InstaPro, Model 600, Des Moines, Iowa), which had a screw compression ratio of 1:1. The die assembly was conical and contained 88 circular 3-mm die holes. The screw speed operated at 600 rpm during extrusion. After extrusion, the pelletized feed blends were dried in a laboratory oven (Thelco Precision, Jovan, Winchester, Virginia) at 50 °C for 24 h. After drying, the diets were broken and sieved into proper pellet size, then stored at −15 °C.

### 2.2. Fecal Collection System, Feeding and Fecal Collection

**Figure 1:**
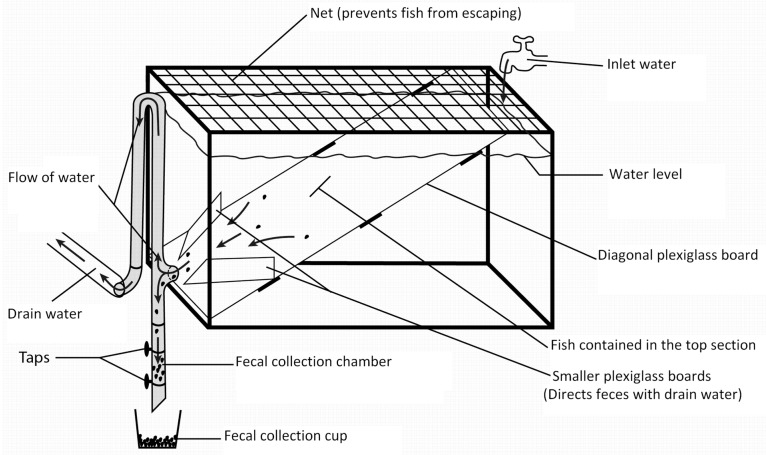
Diagram of a 110 L aquarium tank modified for fecal collection.

Stocker catfish from the UAPB aquaculture research station were collected and moved to the nutrition wet lab for the feeding trial. They were maintained in 240 L aquaria and fed the control diet for 1 week prior to sorting by size and stocking into experimental tanks. Catfish weighing about 150–200 g individually were stocked in each of six 110 L tanks modified for fecal collection at a rate of 10 fish/tank (Figure 1). A plexiglass board was secured diagonally in the tank using silicone, and fish were held in the upper portion. Smaller plexiglass boards were affixed at the water outlet to direct any feces produced into the drain water. Feces that flowed out with the drain water sunk into the fecal collection chamber.

Using a series of two taps, the feces were collected in a plastic cup. The tanks were covered with black sheeting to prevent startling the fish. Each 110 L tank was supplied with dechlorinated municipal tap water. Water flow rate was maintained at 1.1 L/min. Individual air stones aerated each tank. Water temperature was maintained around 26.5 ± 0.3 °C (mean ± SE), which is close to optimum for catfish feed intake [24].

The six diets were assigned randomly to each tank. Fish were fed their respective diets to satiation at 8 am each day for 7 days prior to beginning the fecal collections. Feeding and fecal collection for each diet were conducted in triplicate over time in the following manner. After feeding the fish their respective diets, any uneaten food was drained through the collection chamber prior to fecal collection. Feces production began after 5–6 h. Feces were collected from the fecal collection chamber every 1.5–2.0 h and pooled by tank. All feces were collected and stored in a single container (one per tank).

The collected fecal material was lyophilized using a Labconco Freezone 4.5 freeze-dry system (Labconco Corporation, Kansas City, Missouri) and stored at −20 °C until analysis. After sufficient feces were collected for analysis in the first collection period, diet-tank assignments were re-randomized such that each tank received a different diet for 7 days prior to the second collection period. Fecal collection commenced thereafter, as previously described.

After the second collection period, diets were randomly reassigned as previously described for a third fecal collection period. When incidences of aggression occurred during the study [25], as indicated by external bite marks appearing on the fish, the entire culture system was treated with 2 gm/L sodium chloride to prevent infection. Any severely wounded or dead fish were replaced. After the introduction of a new fish, feeding continued with the respective diets for 2–3 days to allow new fish to acclimate to the new diet. During this period, the feces were discarded to avoid contamination because the newly introduced fish may have consumed other food sources.

### 2.3. Proximate and Fatty Acid Analysis of Diets and Feces

The diets and feces were homogenized prior to analyses. Dry matter and ash were determined according to established procedures [26]. The crude protein in the diets and feces were determined using a Leco Truspec N™ nitrogen analyzer (Leco Inc., St Joseph, MI, USA) at the USDA/ARS–Harry K. Dupree Stuttgart National Aquaculture Research Center, Stuttgart, Arkansas. The results were expressed as g/100 g of total crude protein. Total lipids were extracted from samples and weighed to determine the percentage of lipid [27]. The proximate composition of the finished diets is shown in Table 2.

Lipid extracts were then methylated [28] to produce fatty acid methyl esters (FAME) for fatty acid analysis. The FAME were analyzed with a gas chromatograph equipped with a fused silica capillary column (15 m × 0.25 mm ID; Varian CP select for Fame #CP8510), and a flame ionization detector (Varian, Model CP-3800 fitted with a CP-8200 autosampler, Walnut Creek, CA) using helium as the carrier gas. Injection volume was 1 µL, with an injector and detector temperature of 250 °C and 315 °C, respectively. The column temperature was held initially at 100 °C for 10 min, increased to 160 °C at a rate of 15 °C/min and held for 4 min, then increased to 250 °C at a rate of 2.5 °C/min. Each sample had a total analysis time of 81 min. The FAME in diets and feces were identified and quantified by comparing the retention time and peak area to those of serially diluted mixtures of reference standards (trans-10, cis-12 CLA; cis-9, trans-11 CLA, Matreya LLC, Pleasant Gap, PA; GLC-473b, Nu-Check Prep, Elysian, MN). The results of the individual fatty acids were expressed as g/100 g of total identified FAMEs. The fatty acid composition of the finished diets is shown in Table 3.

### 2.4. Analysis of Inert Marker (Yttrium) and Calculation of Apparent Digestibility Coefficients (ADC)

Concentration of the inert marker, yttrium (Yt), in the diets and the feces was determined using inductively coupled plasma emission spectrometry (ICP-OES; WBA Analytical Laboratories, Springdale, Arkansas). Apparent digestibility coefficients (ADC) of nutrients in the test diets were calculated [4] as follows:

ADC (%) = 100 − 100 [(% Yt in feed)/(% Yt in feces) × (% nutrient in feces/% nutrient in feed)]. Negative digestibility coefficients were adjusted to zero [29].

## 3. Data Analysis

The ADC of crude protein, lipid and fatty acids in the test diets were analyzed by one-way ANOVA using the SAS^®^ 9.2 software program PROC GLM (SAS Institute Inc., Cary, NC, USA) to test for differences among dietary treatments. Data were analyzed with tank as the experimental unit by averaging across each tank. Fisher’s least significance difference test [30] was used to identify specific differences among treatment means with significance declared at *p* ≤ 0.05.

## 4. Results

### 4.1. Fatty Acid Composition of Feces

Saturates (SFA) were higher in feces of fish fed PF or MFO diets compared to the other diets (Table 4). Feces of fish fed the COO diet had a higher concentration of monounsaturates due to elevated levels of 18:1n-9 (24.4%) in COO. Fecal content of 18:2n-6 was consistently high across diets, and it was higher in the CLA-SBO and SBO diets than in other diets.

Feces of fish fed the FXO diet contained the highest concentration of 18:3n-3. The highest concentration of LC-PUFAs, such as 20:5n-3 and 22:6n-3, were found in feces of fish fed the MFO diet. These changes led to a decrease of the n-3/n-6 ratio in feces of fish fed the FXO or MFO diets (1.1 to 0.3 and 0.9 to 0.2, respectively).

### 4.2. Apparent Digestibility Coefficients for Crude Protein, Lipid and Fatty Acids

Crude protein digestibility of the diets ranged from 91.6–92.9%, and there were no differences among diets (*p* = 0.748). The digestibility of total lipids (82.8–88.4%) was also not different among diets (Table 5). However, individual fatty acid digestibilities were affected by dietary lipid source. The digestibility of 14:0 was higher in fish fed the MFO diet (82.3%) than in the PF and COO diets (55.2 and 53.9%, respectively); however, the digestibility of 16:0 and 18:0 were not different among the diets. The digestibility of 16:1n-7 was higher in the MFO (85.7%) and PF (85.7%) diets than in the COO diet (68.5%). The digestibility of 18:1n-9 was lower in fish fed the MFO diets than all other treatments. The CLA isomers *cis*-9, *trans*-11 (84.1%) and *trans*-10, *cis*-12 (90%) were efficiently digested in the CLA-SBO diet, while the digestibility of 18:2n-6 was not different among the diets. The highest digestibility coefficient for 18:3n-3 was observed in fish fed the FXO dietary treatment (90.3%), which had the highest concentration of 18:3n-3. High digestibility coefficients (≥58.5%) were observed for the LC-PUFAs (20:4n-6, 20:5n-3 and 22:6n-3) that were mainly present in the MFO diet (94.2, 93.1, 90.6%, respectively). Arachidonic acid (20:4n-6) was digested more efficiently in fish fed the MFO diet than the COO diet. A dash (no digestibility coefficient shown) signifies that the computation of digestibility coefficients was not possible because these fatty acids were detected in the initial diets and not detected in the feces, or vice versa. No coefficients could be generated for the n-3 LC-PUFA in fish fed diets other than MFO, so statistical comparisons were not possible.

## 5. Discussion

Fatty acid composition of the diet is the main determinant of lipid digestibility in fish, though lipid class composition is also a factor [31]. The CLA-enhanced soybean oil and the other dietary lipid sources did not affect lipid or protein digestibility in Channel Catfish, which also occurred in Australian Shortfin Eel [32] and Red Hybrid Tilapia [33]. However, lipid digestibility was higher in Atlantic Halibut-fed vegetable oils (93–95%) compared to those fed animal lipids (90%) [9], which was attributed to the high concentration of SFA in the animal lipids. Lipid digestibility decreased with increasing PUFA to LC-PUFA ratio in European Sea Bass [34]. The degree of unsaturation, carbon chain length and the concentration of fatty acid in the lipid can all influence fatty acid digestibility [9,33,35,36]. Saturated fatty acids have a higher melting point than unsaturated fatty acids of the same chain length, resulting in lower digestibility of SFA in fish [37]. Endogenous fatty acid secretions in catfish in this study were observed for fatty acids present in low dietary concentrations. For example, 14:0 was not present in the CLA-SBO diet, and dietary concentrations for the SBO and FXO diets were 0.1 and 0.13%, respectively; whereas fecal concentrations of 14:0 from fish fed those diets were much higher (0.6–0.9%). The current data might suggest that endogenous fatty acids were secreted to compensate for fatty acids that were present in low dietary concentrations. Alternatively, the digestibility of certain fatty acid groups may be underestimated due to the presence of endogenously produced fatty acids, as previously suggested in Rainbow Trout [37]. Endogenously produced fatty acids can affect the computation of digestibility coefficients of fatty acids, leading to very low or negative computed coefficients. This can occur when fatty acids are present at low dietary concentrations and have low digestibility [4].

In this study, the digestibility of SFA and other fatty acids was not dependent on the concentration of dietary SFA. The digestibility of SFA was below 61% and lower than MUFA, which had a digestibility of between 64 to 78%, while the digestibility for n-3 and n-6 fatty acids was between 53 to 91% and 55 to 70%, respectively. These results are contrary to observations in other fish species. For example, the digestibility of SFA in Rainbow Trout-fed diets formulated from a combination of capelin or anchovy oil with soybean, rapeseed, palm or olive oil decreased with increasing dietary concentration of SFAs [38]. The digestibility of MUFA, n-3 and n-6 fatty acids also decreased with increasing SFA, but to a lesser extent. High dietary concentrations of SFA also reduced the digestibility of SFA in Atlantic Salmon [13]. However, SFA had little effect on the digestibility of SFA and other fatty acid groups in Atlantic Salmon fed diets with soybean oil with a low concentration of SFA [39].

Interestingly, pigs have a similar gut morphology to Channel Catfish [4,40]. Jørgensen, et al., [41] fed pigs (35 kg) a basal diet or diets containing 15% of either fish oil, rapeseed oil or coconut oil. All the diets contained 22% fish meal as a protein source. The higher ileal digestibility of 18:1 and 18:3n-3 was related to the higher dietary concentration of these fatty acids in diets with fish oil and rapeseed oil, as well as higher 18:2n-6 in the rapeseed oil diets. Moreover, the digestibility of n-3 LC-PUFAs was also high in all the swine diets. The negative digestibility coefficient of 18:0 in the swine trial was attributed to the low dietary concentration, low intake and endogenous gut secretions. Similarly, in growing pigs (40 kg) fed diets containing 10% of either tallow, high oleic sunflower oil, sunflower oil, linseed oil or a fat blend of tallow (5.5%), sunflower oil (3.5%) and 1% linseed oil [42], the ileal digestibility of 18:3n-3 in the linseed oil diet and 18:2n-6 in the sunflower oil diet was higher in the diets in which they were present in the highest concentrations. However, the digestibilities of 16:0 and 18:0 were lower in pigs fed the tallow diet, even though the dietary concentration of these fatty acids was higher in this diet. The lower digestibility of 18:0 was determined to be due to the use of dietary animal fats in pigs [43].

In the present study, the digestibility of n-3 fatty acids was positively correlated with dietary concentration. The 18:3n-3 and n-3 LC-PUFA were well absorbed from the FXO and MFO diets, respectively, in which they were found in higher proportions. The digestibility of 18:3n-3 was highest in fish fed the FXO diet (90.3%), and digestibility coefficients of 20:5n-3, 22:5n-3 and 22:6n-3 were high (90–100%) in fish fed the MFO diet. Higher digestibility of 18:3n-3 and n-3 LC-PUFA also occurred in Atlantic Halibut with the inclusion of flaxseed oil and fish oil, respectively, in the diets [9]. In cod, lipase specificity was also high for 20:5n-3, 20:4n-6 and 18:2n-6 compared to 22-carbon fatty acids, SFA and MUFA (except 18:1n-9) [44]. Lipolysis is a rate-limiting step in fatty acid digestion and the rate of release from triacylglycerol is higher for PUFA [45] than for 18:0 and MUFA. Lipolytic activity in juvenile turbot indicated preferential absorption of PUFA over MUFA, followed by SFA as digesta moved from the stomach to the rectum [46].

We observed some interesting trends in fecal fatty acids that suggested differences in metabolism mediated by key dietary fatty acids. For instance, there were no n-3 LC PUFA in either the SBO or CLA-SBO diets, yet both 20:5n-3 and 22:6n-3 appeared in the feces of fish fed the CLA-SBO diet only. Increased 20:5n-3 and 22:6n-3 has been observed in tissues of other freshwater fish fed diets with CLA [47,48,49], indicating increased delta-6 desaturase activity. The COO diet also lacked n-3 LC PUFA, yet feces of catfish fed that diet contained 22:6n-3. The low level of 18:3n-3 present in the diet (3%) appeared to stimulate delta-6 desaturase activity and production of n-3 LC PUFA. In contrast, fish fed the FXO diet (with no LC PUFA but about 34% 18:3n-3) produced feces without LC-PUFA. We did not measure desaturase activity in our study. However, delta-6 desaturase is the rate-limiting enzyme in LC-PUFA synthesis, and the balance between n-3 and n-6 fatty acids affects the amounts and types of LC-PUFA produced. Catfish can convert 18:3n-3 into n-3 LC-PUFA, and fish fed the FXO diet had the highest ADC for 18:3n-3. The lack of LC-PUFA in feces of those fish might be explained by reduced desaturase activity. These speculations can be addressed with more detailed studies of catfish lipid metabolism that include measurements of desaturase activity.

It is difficult to reconcile differences in the results of lipid digestibility studies of different fish species, because they cover a wide range of trophic levels (herbivorous, omnivorous and carnivorous). Experimental and commercial diets for fish range widely in nutrient composition as a result. Diets for omnivorous Channel Catfish rarely contain more than 10% total lipid, whereas those for carnivorous species can exceed 25% [4]. The EFA requirements also vary considerably—omnivorous fish such as catfish can synthesize LC-PUFA from PUFA, whereas carnivorous fish generally require preformed LC-PUFA in the diet.

Fish behavior can also affect feed utilization. For instance, stress can reduce feed intake, though specific effects on nutrient digestibility are not well documented. The aggressive behavior noted in catfish in this study sometimes reduced feed intake. However, fish that were not actively feeding were replaced, and no differences in behavior were observed among diets. Aggression in catfish held in aquaria has been noted previously [50]. There is no specific stocking density that will prevent aggressive behavior in catfish; it tends to escalate with fish size, and larger fish are preferred for digestibility trials to ensure sufficient production of feces.

Research demonstrating the benefits of plant oils in catfish diets is now substantial, which is likely to result in increased inclusion in commercial diets. The CLA isomers in CLA-Soy oil were well-digested in catfish in this study. Previous results showed that CLA-Soy oil also promotes overall catfish performance, does not compromise the sensory characteristics of the fillet and is beneficial for human health. The combined results indicate the unique potential of CLA-Soy oil to contribute to the sustainability of Channel Catfish production.

## 6. Conclusions

The efficient absorption of 18:3n-3 and n-3 LC-PUFAs from the FXO and MFO diets, respectively, is in agreement with the n-3 EFA requirements of Channel Catfish (Satoh, et al., 1989). Substitution of fish oils with plant oils in aquatic animal diets is likely to increase due to concerns over environmentally sustainable production practices, including diet composition. The CLA-soybean oil is a novel plant lipid with good potential to enhance healthy fatty acids in Channel Catfish because of its high digestibility compared to other lipid sources. In particular, the high digestibility coefficient of CLA isomers *cis*-9, *trans*-11 (84.1%) and *trans*-10, *cis*-12 (90%) confirms that they were efficiently absorbed from the CLA-SBO diet.

## Figures and Tables

**Table 1:** Basal diet formulation (%; as fed) used in a lipid digestibility trial with Channel Catfish.

Ingredients	%
Soybean meal ^1^	50.0
Cottonseed meal ^1^	10.0
Wheat shorts ^1^	31.0
Yttrium Oxide ^2^	0.5
Vitamin premix ^3^	1.0
Mineral premix ^3^	1.0
Ethoxyquin	0.0125
Supplemental lipid ^4^	4.0
Carboxymethylcellulose	2.5

^1^ Dehulled, solvent-extracted soybean meal (48% crude protein), solvent-extracted cottonseed meal (41.7% crude protein) and wheat shorts (17% crude protein) [3]. ^2^ Yttrium oxide was purchased from Sigma-Aldrich Corp., St. Louis, Missouri. ^3^ Same as [23]. ^4^ CLA-soybean oil, soybean oil, catfish offal oil, flaxseed oil, menhaden fish oil or poultry fat.

**Table 2:** Mean proximate composition (%; dry weight basis) of experimental diets supplemented with 4% (by weight) of either soybean oil (SBO), SBO enriched with conjugated linoleic acids (CLA-SBO), catfish offal oil (COO), flaxseed oil (FXO), menhaden fish oil (MFO) or poultry fat (PF), and fed to Channel Catfish in a digestibility trial ^1^.

Diet	Lipid	Protein	Dry Matter	Ash
SBO	8.2	32.9	93.4	7.6
CLA-SBO	7.6	32.2	93.6	7.8
COO	7.9	32.3	95.8	7.4
FXO	7.3	32.7	93.8	7.3
MFO	7.3	32.4	93.3	7.1
PF	7.7	33.0	95.1	7.3

^1^ Values are means of duplicate analyses of each diet.

**Table 3:** Mean (±SE) fatty acid composition (% of total fatty acids by weight) of diets supplemented with 4% of either soybean oil (SBO), SBO enriched with conjugated linoleic acids (CLA-SBO), catfish offal oil (COO), flaxseed oil (FXO), menhaden fish oil (MFO) or poultry fat (PF) ^1^.

	Diets					
FATTY ACID	SBO	CLA-SBO	COO	FXO	MFO	PF
14:0	0.1 ± 0.1	ND	0.8 ± 0.03	0.1 ± 0.002	5.7 ± 0.1	0.7 ± 0.04
16:0	14.7 ± 0.4	16.1 ± 0.2	19.3 ± 0.02	11.1 ± 0.1	19.7 ± 0.1	22.7 ± 0.1
18:0	4.0 ± 0.004	4.7 ± 0.01	5.0 ± 0.01	4.0 ± 0.02	3.8 ± 0.02	5.0 ± 0.02
20:0	0.3 ± 0.01	0.4 ± 0.02	ND	ND	ND	ND
22:0	0.4 ± 0.01	0.4 ± 0.004	ND	ND	ND	ND
∑ SFA ^2^	19.4 ± 0.3	21.6 ± 0.1	25.0 ± 0.1	15.3 ± 0.1	29.2 ± 0.1	28.4 ± 0.2
16:1n-7	0.1 ± 0.1	0.1 ± 0.1	1.8 ± 0.001	ND	7.1 ± 0.02	4.9 ± 0.02
18:1n-9	20.6 ± 0.1	22.7 ± 0.1	36.2 ± 0.02	19.2 ± 0.2	12.0 ± 0.01	30.7 ± 0.2
18:1n-7	1.5 ± 0.02	1.6 ± 0.05	1.7 ± 0.05	0.8 ± 0.02	2.6 ± 0.02	1.8 ± 0.01
20:1n-9	0.1 ± 0.1	0.5 ± 0.002	0.9 ± 0.004	0.1 ± 0.1	0.4 ± 0.01	0.1 ± 0.12
22:1n-11	ND	ND	ND	ND	1.2 ± 0.002	ND
∑ MUFA ^3^	22.2 ± 0.1	24.9 ± 0.1	40.6 ± 0.03	20.1 ± 0.3	23.3 ± 0.01	37.5 ± 0.3
CLA-9c,11t	ND	1.0 ± 0.03	ND	ND	ND	ND
CLA-10t,12c	ND	0.9 ± 0.01	ND	ND	ND	ND
∑CLA isomers ^4^	ND	1.9 ± 0.04	ND	ND	ND	ND
18:2n-6	52.7 ± 0.1	47.0 ± 0.1	30.6 ± 0.1	30.7 ± 0.2	24.6 ± 0.2	31.2 ± 0.1
18:3n-3	5.6 ± 0.04	4.5 ± 0.01	3.0 ± 0.03	33.9 ± 0.4	4.4 ± 0.1	2.9 ± 0.04
20:2n-6	ND	ND	0.5 ± 0.003	ND	ND	ND
20:4n-6	ND	ND	0.3 ± 0.02	ND	0.7 ± 0.0	ND
20:5n-3	ND	ND	ND	ND	8.2 ± 0.04	ND
22:5n-3	ND	ND	ND	ND	1.3 ± 0.01	ND
22:6n-3	ND	ND	ND	ND	8.3 ± 0.002	ND
∑ n-3 ^5^	5.6 ± 0.04	4.5 ± 0.01	3.0 ± 0.03	33.9 ± 0.4	22.3 ± 0.1	2.9 ± 0.04
∑ n-6 ^6^	52.7 ± 0.1	47.0 ± 0.1	31.4 ± 0.1	30.7 ± 0.2	25.3 ± 0.2	31.2 ± 0.1
∑ n-3 LC-PUFA ^7,8^	ND	ND	ND	ND	17.8 ± 0.03	ND
∑ n-6 LC-PUFA ^7,9^	ND	ND	0.8 ± 0.04	ND	0.7 ± 0.0	ND
n-3/n-6	0.1 ± 0.001	0.1 ± 0.0001	0.1 ± 0.001	1.1 ± 0.02	0.9 ± 0.007	0.1 ± 0.001

^1^ Fatty acid analysis of diets was conducted in duplicate. ND, fatty acid that could not be detected. ^2^ Total saturates (SFA) included 14:0, 16:0, 18:0, 20:0 and 22:0. ^3^ Total monounsaturates (MUFA) included 16:1, 18:1, 20:1 and 22:1. ^4^ Total CLA isomers included 9c, 11t and 10t, 12c. ^5^ Total n-3 fatty acids included 18:3n-3, 20:5n-3, 22:5n-3 and 22:6n-3. ^6^ Total n-6 fatty acids included 18:2n-6 (excluding CLA isomers), 20:2n-6 and 20:4n-6. ^7^ Long chain polyunsaturated fatty acids (LC-PUFAs). Fatty acids with 20 or more carbons and four or more double bonds. ^8^ Total n-3 LC-PUFAs included 20:5n-3, 22:5n-3 and 22:6n-3. ^9^ Total n-6 LC-PUFAs included 20:2n-6 and 20:4n-6.

**Table 4:** Selected mean (±PSE) fatty acid composition (% of total fatty acids by weight) of feces from Channel Catfish fed diets supplemented with 4% of either soybean oil (SBO), SBO enriched with conjugated linoleic acids (CLA-SBO), catfish offal oil (COO), flaxseed oil (FXO), menhaden fish oil (MFO), or poultry fat (PF) ^1^.

	Diets							
Fatty Acid	SBO	CLA-SBO	COO	FXO	MFO	PF	PSE ^2^	Pr > F
14:0	0.7 bc	0.6 c	1.0 b	0.9 bc	3.2 a	0.9 bc	0.1	<0.001
16:0	20.0 d	20.5 cd	22.7 bc	22.3 cd	25.0 b	30.0 a	0.9	<0.001
18:0	5.7	6.2	6.4	6.9	6.2	7.6	0.6	0.388
20:0	0.4	0.5	0.4	0.3	0.4	0.3	0.06	0.213
22:0	0.6 a	0.6 a	0.4 b	0.5 b	0.5 b	0.3 c	0.04	0.001
∑ SFA ^3^	27.4 b	28.5 b	30.9 b	30.9 b	35.3 a	39.1 a	1.4	<0.001
16:1n-7	0.8 d	1.0 cd	1.4 c	0.6 d	3.2 a	2.1 b	0.2	<0.001
18:1n-9	18.2 b	18.6 b	24.4 a	18.8 b	15.6 c	20.3 b	0.8	<0.001
18:1n-7	2.1 b	2.1 b	2.3 b	2.2 b	2.8 a	2.1 b	0.1	0.019
20:1n-9	0.5 b	0.5 b	0.8 a	0.5 b	0.5 b	0.4 b	0.04	<0.001
22:1n-11	ND	0.1	ND	ND	0.1	0.1	0.1	0.690
∑ MUFA ^4^	21.5 c	22.5 c	29.0 a	22.1 bc	22.3 bc	25.2 b	1.03	0.002
CLA-9c,11t	ND	0.5	ND	ND	ND	ND	0.03	-----
CLA-10t,12c	ND	0.3	ND	ND	ND	ND	0.03	-----
∑CLA isomers ^5^	ND	0.8	ND	ND	ND	ND	0.05	-----
18:2n-6	45.6 a	43.6 a	35.6 bc	37.0 b	34.1 bc	32.0 c	1.2	<0.001
18:3n-3	4.7 b	4.3 bc	3.4 d	9.9 a	3.8 cd	3.1 d	0.3	<0.001
20:2n-6	0.2	0.1	0.4	0.1	0.1	0.1	0.1	0.056
20:4n-6	0.2	0.2	0.3	ND	0.1	0.2	0.1	0.876
20:5n-3	ND	0.1 b	ND	ND	1.8 a	ND	0.1	<0.001
22:5n-3	ND	ND	ND	ND	ND	ND	-----	-----
22:6n-3	ND	0.2 b	0.3 b	ND	2.4 a	ND	0.2	<0.001
∑ n-3 ^6^	4.7 c	4.7 c	3.7 cd	9.9 a	8.0 b	3.1 d	0.3	<0.001
∑ n-6 ^7^	46.0 a	43.9 a	36.3 bc	37.1 b	34.3 bc	32.7 c	1.2	<0.001
∑ n-3 LC-PUFA ^8,9^	ND	0.3 b	0.3 b	ND	4.2 a	ND	0.3	<0.001
∑ n-6 LC-PUFA ^8,10^	0.4	0.2	0.9	0.1	0.2	0.3	0.2	0.081
n-3/n-6	0.1 c	0.1 c	0.1 c	0.3 a	0.2 b	0.1 c	0.006	<0.001

^1^ Values are means of three replicates per treatment analyzed in duplicate; ND denotes that the fatty acid was not detected; a dash denotes that the indicated statistic could not be computed. Means within rows with different letters are significantly different (*p* ≤ 0.05). Fisher’s least significance difference test was used to identify specific differences. ^2^ Pooled standard error (PSE). ^3^ Total saturates (SFA) included 14:0, 16:0, 18:0, and 20:0. ^4^ Total monounsaturates (MUFA) included 16:1, 18:1, 20:1, and 22:1. ^5^ Total CLA isomers included 9c, 11t; and 10t, 12c. ^6^ Total n-3 fatty acids included 18:3n-3, 20:5n-3, 22:5n-3, and 22:6n-3. ^7^ Total n-6 fatty acids included 18:2n-6 (excluding CLA isomers), 20:2n-6 and 20:4n-6. ^8^ Long-chain polyunsaturated fatty acids (LC-PUFA). Fatty acids with 20 or more carbons and four or more double bonds. ^9^ Total n-3 LC-PUFA included 20:5n-3, 22:5n-3, and 22:6n-3. ^10^ Total n-6 LC-PUFA included 20:2n-6 and 20:4n-6.

**Table 5:** Mean apparent digestibility coefficients (ADCs; %) of lipid and fatty acids for Channel Catfish fed diets supplemented with 4% of either soybean oil (SBO), SBO enriched with conjugated linoleic acids (CLA-SBO), catfish offal oil (COO), flaxseed oil (FXO), menhaden fish oil (MFO), or poultry fat (PF) ^1^.

	Diets							
Fatty Acid	SBO	CLA-SBO	COO	FXO	MFO	PF	PSE ^2^	Pr > F
lipid	83.0	82.8	83.9	83.5	88.4	84.9	3.7	0.889
14:0	0.0 c	-----	53.9 b	0.0 c	82.3 a	55.2 b	2.8	<0.001
16:0	50.1	57.7	53.8	33.4	59.2	55.9	7.0	0.178
18:0	46.4	55.4	48.2	41.9	47.1	49.4	9.9	0.957
20:0	52.7	57.7	-----	-----	-----	-----	4.7	0.496
22:0	40.6	47.5	-----	-----	-----	-----	6.4	0.489
∑SFAs ^3^	48.2	56.0	51.3	33.6	60.9	54.1	7.5	0.238
16:1n-7	0.0 c	0.0 c	68.5 b	-----	85.7 a	86.0 a	1.8	<0.001
18:1n-9	67.8 b	72.8 ab	73.9 ab	68.1 b	58.4 c	78.0 a	2.9	0.007
18:1n-7	46.9 b	57.2 ab	48.6 b	0.0 c	66.0 a	61.1 ab	5.6	<0.001
20:1n-9	0.0 c	67.0 a	66.0 a	0.0 c	58.2 b	0.0 c	2.4	<0.001
22:1n-11	-----	-----	-----	-----	97.1	-----	2.8	-----
∑MUFAs ^4^	64.7	70.3	72.3	64.5	69.3	77.8	3.0	0.073
CLA-9c,11t	-----	84.1	-----	-----	-----	-----	1.8	-----
CLA-10t,12c	-----	90.0	-----	-----	-----	-----	0.9	-----
∑CLAisomers ^5^	-----	86.9	-----	-----	-----	-----	1.1	-----
18:2n-6	68.6	69.4	55.3	60.3	55.6	65.3	3.9	0.081
18:3n-3	69.9 bc	68.3 bc	56.5 d	90.3 a	73.0 b	64.3 c	2.5	<0.001
20:2n-6	-----	-----	72.3	-----	-----	-----	1.8	-----
20:4n-6	-----	-----	58.5 b	-----	94.2 a	-----	6.6	0.019
20:5n-3	-----	-----	-----	-----	93.1	-----	0.2	-----
22:5n-3	-----	-----	-----	-----	100.0	-----	0.0	-----
22:6n-3	-----	-----	-----	-----	90.6	-----	0.9	-----
∑n-3 ^6^	70.0 b	70.0 b	53.5 c	90.3 a	88.5 a	64.3 b	2.6	<0.001
∑n-6 ^7^	68.3	68.3	55.4	60.5	56.3	65.0	3.9	0.103
∑n-3 LC-PUFA ^8,9^	-----	-----	-----	-----	92.3	-----	0.5	-----
∑n-6 LC-PUFA ^8,10^	-----	-----	66.0 b	-----	94.2 a	-----	5.0	0.016

^1^ Values are means of three replicates per treatment; a dash denotes that the ADC or ANOVA *p*-value could not be computed. Means within rows with different letters are significantly different (*p* ≤ 0.05). Fisher’s least significance difference test was used to identify specific differences. ^2^ Pooled standard error (PSE). ^3^ Total saturates (SFA) included 14:0, 16:0, 18:0, and 20:0. ^4^ Total monounsaturates (MUFA) included 16:1, 18:1, 20:1, and 22:1. ^5^ Total CLA isomers included 9c, 11t; and 10t, 12c. ^6^ Total n-3 fatty acids included 18:3n-3, 20:5n-3, 22:5n-3, and 22:6n-3. ^7^ Total n-6 fatty acids included 18:2n-6 (excluding CLA isomers), 20:2n-6 and 20:4n-6. ^8^ Long-chain polyunsaturated fatty acids (LC-PUFA). Fatty acids with 20 or more carbons and four or more double bonds. ^9^ Total n-3 LC-PUFA included 20:5n-3, 22:5n-3, and 22:6n-3. ^10^ Total n-6 LC-PUFA included 20:2n-6 and 20:4n-6.

## Data Availability

Data collected is available upon reasonable request from the authors of this study.
